# Artificial Intelligence-Based Methods for Precision Cardiovascular Medicine

**DOI:** 10.3390/jpm13081268

**Published:** 2023-08-16

**Authors:** Farida Mohsen, Balqees Al-Saadi, Nima Abdi, Sulaiman Khan, Zubair Shah

**Affiliations:** College of Science and Engineering, Hamad Bin Khalifa University, Qatar Foundation, Doha 34110, Qatar

**Keywords:** precision medicine, cardiovascular diseases, artificial intelligence, machine learning

## Abstract

Precision medicine has the potential to revolutionize the way cardiovascular diseases are diagnosed, predicted, and treated by tailoring treatment strategies to the individual characteristics of each patient. Artificial intelligence (AI) has recently emerged as a promising tool for improving the accuracy and efficiency of precision cardiovascular medicine. In this scoping review, we aimed to identify and summarize the current state of the literature on the use of AI in precision cardiovascular medicine. A comprehensive search of electronic databases, including Scopes, Google Scholar, and PubMed, was conducted to identify relevant studies. After applying inclusion and exclusion criteria, a total of 28 studies were included in the review. We found that AI is being increasingly applied in various areas of cardiovascular medicine, including the diagnosis, prognosis of cardiovascular diseases, risk prediction and stratification, and treatment planning. As a result, most of these studies focused on prediction (50%), followed by diagnosis (21%), phenotyping (14%), and risk stratification (14%). A variety of machine learning models were utilized in these studies, with logistic regression being the most used (36%), followed by random forest (32%), support vector machine (25%), and deep learning models such as neural networks (18%). Other models, such as hierarchical clustering (11%), Cox regression (11%), and natural language processing (4%), were also utilized. The data sources used in these studies included electronic health records (79%), imaging data (43%), and omics data (4%). We found that AI is being increasingly applied in various areas of cardiovascular medicine, including the diagnosis, prognosis of cardiovascular diseases, risk prediction and stratification, and treatment planning. The results of the review showed that AI has the potential to improve the performance of cardiovascular disease diagnosis and prognosis, as well as to identify individuals at high risk of developing cardiovascular diseases. However, further research is needed to fully evaluate the clinical utility and effectiveness of AI-based approaches in precision cardiovascular medicine. Overall, our review provided a comprehensive overview of the current state of knowledge in the field of AI-based methods for precision cardiovascular medicine and offered new insights for researchers interested in this research area.

## 1. Introduction

Globally, cardiovascular diseases (CVDs) are well-known major causes of mortality, accounting for nearly one-third of all deaths in the world [1]. In the United States (US), CVDs are widespread, with one in three adults having some type of CVDs [2], and the number of cases has doubled to approximately 523 million worldwide [3]. In 2035, about half of the US population is expected to suffer from at least one kind of CVDs [4]. The shift from population-based care toward more patient-centred approaches in healthcare has been accompanied by a shift in the management of disease processes. One aspect is a greater emphasis on precision medicine (PM). PM is an emerging healthcare model that takes into account individual variations in lifestyles, genes, and environments [5,6].

Precision cardiovascular medicine aims to optimize the diagnosis, risk prediction, prognostication, and therapeutic intervention by integrating large multimodal biomedical datasets incorporating individual genes, function, lifestyle, and environmental variations [7]. The significant benefit of this approach over conventional medical methods is its use of high-dimensional datasets to determine an individual’s health status, treatment response, and prognosis. The data can be obtained from various sources such as electronic health records (EHR), genomics and other multi-omics data, medical imaging, wearable sensors, biosensors, and behavioural monitors. Through the application of precision cardiovascular medicine, diagnostic, predictive, and therapeutic errors could be significantly reduced. As an example, by interrogating longitudinal medical datasets, one can identify disease subtypes and match the patient to those with similar disease profiles; through the knowledge of treatment effectiveness and outcomes, the prognosis of this patient would be more precise, and therapy recommendations would be optimized in accordance with similar subgroups [8].

AI methods, such as deep learning (DL) models and machine learning (ML), enable the integration of complex multimodal biomedical datasets to generate multimodal insights to facilitate precision medicine [9]. In fact, CVDs are complex and heterogeneous in nature, as they are caused by multiple genetic, environmental, and behavioural factors [10]. Therefore, AI algorithms can potentially find the cryptic and genotypic structures to be used in advanced patient care, such as diagnosing the disease early, predicting the treatment response, predicting the risk of developing the disease in the future, prognosis, and other outcomes in individual patients [5]. Lately, researchers have developed personalized prediction models in cardiology. Chaves et al. [11] proposed a DL-based framework for opportunistic risk assessment of ischemic heart disease (IHD) using medical imaging data combined with patient EHR. Zhao et al. modelled genetic data fused with EHR for practising the the 10-year risk of IHD [12].

The integration of AI in PM will revolutionize cardiovascular healthcare delivery. To this end, this review aimed to provide a comprehensive review of recent developments and the use of AI methods and their applications for PM in cardiovascular medicine. There are some other reviews in the literature that focus on the use of AI for cariology [13,14,15,16]; however, we differed from them in terms of the scope and coverage of our review. Some previous reviews focused on the use of AI for specific CVD diseases, such as cardiac arrest [13] and acute coronary syndrome [16]; they did not cover all cardiology diseases. Other reviews focused on paediatric cardiology using AI; Van et al. reviewed AI applications in paediatric cardiology from 2020 to the present and summarized the foundational work and incremental progress [14]. To the best of our knowledge, there is no review paper covering AI models for CVD precision medicine. To this end, our review summarized studies relating to AI-powered PM-based diagnosis, risk prediction, treatment selection, and prognosis of any CVD.

The main purpose of our scoping review was to analyse and synthesise the scientific literature that uses AI models for different precision cardiovascular medicine applications answering four main questions:

- PM branches (applications): What types of PM clinical applications are addressed using AI-based models for CVDs?
- CVD types: For what type of CVDs are AI-powered PM models implemented?
- AI models: What AI algorithms are most commonly applied for different PM applications in CVDs?
- Data sources: What are the medical data modalities used for each model? What are the most commonly used datasets?

This review provided researchers and professionals in the healthcare domain with a comprehensive overview of the advancements made in precision cardiovascular medicine using AI-based methods. Additionally, the study offered a list of publicly available CVDs-related medical datasets that could help AI researchers to develop innovative research methods.

## 2. Methods

We conducted a literature search in famous databases and conducted a scoping review of the existing literature on the applications of AI for cardiovascular precision medicine. We followed the guidelines recommended by the PRISMA-ScR (Preferred Reporting Items for Systematic Reviews and Meta-Analyses Extension for Scoping Reviews) [17].

### 2.1. Search Strategy

#### 2.1.1. Search Sources

This review searched three different databases: Scopus, PubMed, and Google Scholar. It is worth noting that PubMed includes MEDLINE. In Google Scholar, we selected the first 100 relevant studies, as beyond 100 entries, the search results lost relevance and were unrelated to our review. Besides searching the three databases, the reference lists of the included studies were also screened to obtain further pertinent studies.

#### 2.1.2. Search Terms

A literature review and a consultation with experts in the field enabled us to define search terms. Our study focused on studies that used AI models for cardiovascular precision medicine; therefore, the search string was a combination of three search terms connected by AND: (“cardiovascular disease” AND “artificial intelligence” AND “precision medicine”). Different forms of each term were used. The detailed search string used for each database is shown in Supplementary File S1.

### 2.2. Search Eligibility Criteria 

In this review, we included all studies applying AI/ML methods to perform population phenotyping, risk stratification, disease diagnosis, early prediction, mortality and survival prediction, treatment prediction, or therapy efficiency prediction for any CVD disease. We did not restrict the types of AI methods, the type of CDV disease, the used medical data modalities, or the type of clinical outcomes. Moreover, no restrictions were placed regarding age, gender, and ethnicity. We included peer-reviewed studies, book chapters, and conference proceedings. The studies included in this review were all limited to English only. 

We excluded studies that did not focus on CVDs and did not use AI-based models. Non-English studies, Conference abstracts, reviews, commentaries, letters to editors, and animal studies were also excluded. 

### 2.3. Study Selection

Rayyan web-based review management [18] tool was used for the first screening and study selection. Two reviewers, BA and NB, independently performed two phases of screening. The first screening involved assessing study titles and abstracts and removing duplicates. In the second screening phase, the full text was examined against the predefined eligibility criteria to perform study selection. Disagreements between the two reviewers were resolved through discussion. A third author (FM) was consulted when an agreement could not be reached.

### 2.4. Data Extraction 

To ensure a consistent and accurate data extraction process, we developed a form and tested it on a sample of five studies. The extracted data included the name of the first author, publication year, country of the first author’s institution, type of cardiovascular disease, clinical outcome (precision medicine branch), data modalities, data source, and AI models. The data extraction was performed independently by two reviewers (BA and NB), and any disagreements were resolved through discussion, with a third author (FM) consulted as needed.

### 2.5. Data Synthesis

We used a narrative approach to synthesise the data after the data extraction. We analysed the studies from three perspectives: CVD disease branch, PM branch (diagnosis, prediction, phenotyping, etc.), AI models, and data sources/type and size. We used MS Excel for performing and managing data synthesis. A summary of all the data extracted from included studies is given in Supplementary File S2. 

## 3. Results

### 3.1. Search Results

**Figure 1:**
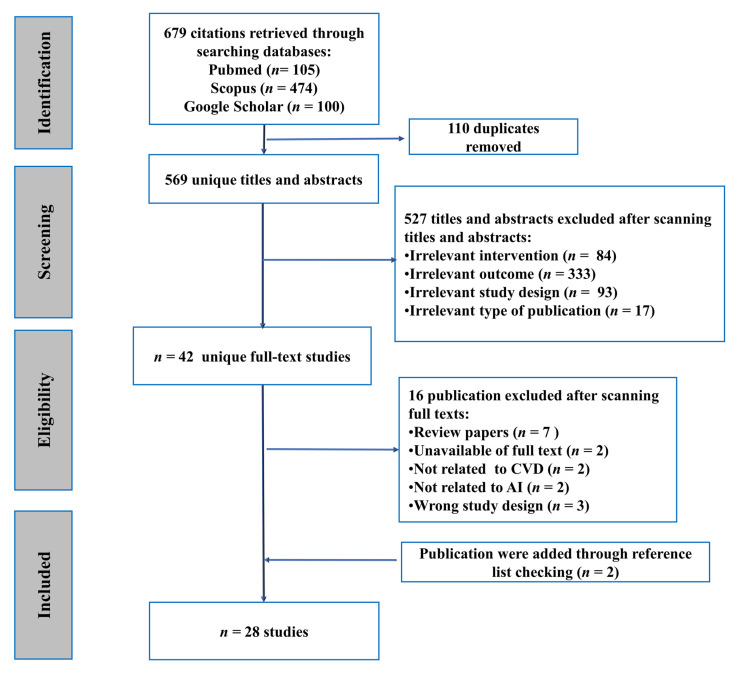
Flowchart diagram of the study selection process.

Our search initially identified 679 studies, of which 569 were retained after removing duplicates. Of these, 42 studies were selected for full-text review based on the inclusion criteria outlined in the Section 2. After further review, 14 studies were excluded, while 2 additional studies were identified by checking reference lists. In total, 28 studies met the inclusion criteria and were included in the data extraction and synthesis process. The study screening and selection process is summarized in Figure 1.

### 3.2. Demographics of the Studies

After extracting the enriched information from the finalized relevant articles, it was concluded that most of the articles are journal articles (*n* = 26). This high number of journal articles reflects the interest of the researchers to work in this research domain. In addition, it was observed that most of the work was reported from the USA, UK, China, Netherlands, and other developed countries, but no work was reported from the third countries. Moreover, the results are synthesised for a comparatively smaller dataset comprising 1000 samples for training and testing purposes. Only four articles used a sample size of more than 20,000 scanned MRI/CT scan images. Table 1 shows the demographics of the included studies of this review.

### 3.3. Cardiovascular Disease Branch

**Figure 2:**
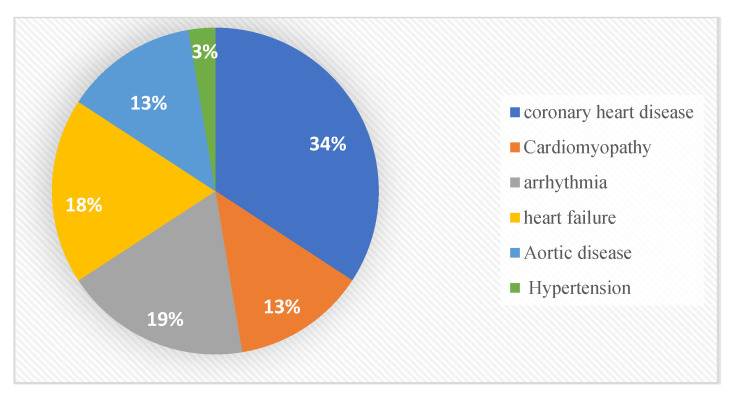
CVD distribution in the included studies.

Different types of cardiovascular diseases (CVD) are identified after analysing the accumulated relevant articles, as depicted in Figure 2. These CVD types are classified into six categories including coronary heart disease [19,20,21,22,23,24,25,26,27,28,29], heart failure [22,26,30,31,32,33,34], arrhythmia [29,33,35,36,37,38,39], aortic diseases [25,26,29,40,41], and cardiomyopathy [26,29,33,42,43], and only one study for hypertension (HTN) [44]. Among these finalised 28 articles, the most represented category is coronary heart disease (*n* = 14). This high contribution is mostly relevant to chronic diseases and represents the complications and high mortalities. This mortality factor can be minimised if people at high risk can be diagnosed well before an incident of a coronary event [21].

### 3.4. Precision Medicine Branch

In this research, the use of AI in precision medicine for CVD was classified into four different branches. These branches were prediction, diagnosis, phenotyping, and risk stratification. All these branches were detailed in the following subsections of the paper.

#### 3.4.1. Prediction

Disease prediction uses data to feed algorithms to predicate disease occurrence or the effectiveness of specific medication and other purposes [45]. In this review, 14 papers out of 28 contributed studies on the purpose of prediction [19,20,21,22,23,24,25,26,27,28,29,46]. These papers were further classified into early prediction, mortality prediction, disease prediction, and dose prediction.

- **Early prediction**—In these 14 articles, 3 studies conducted early detection [25,30,35]. One study was conducted to develop a platform that can detect arrhythmias in real-time using the electrocardiogram (ECG) signal from the patient’s records. As a gradual optimization process, the artificial bee colony (ABC) technique detects and classifies different ECG signals using least-square twin support vector machines (LSTSVMs). As a result of this study, the algorithm achieved a high accuracy and sensitivity rate, indicating that it was a success and will aid in detecting arrhythmias early [35]. Another study reported on determining the pretest probability of coronary artery disease (CAD) and how to continue further in the diagnostic and therapeutic process [25]. The modality data collected from EHR are clinical, pathological, familial, pharmacological history, and lifestyle habits, besides the proteomics omics data. Combining these two modalities of data showed that a panel of 50 proteins outperforms the clinical risk model in predicting the risk of myocardial infarction, and a Gradient boosting classifier algorithm was applied for this study [25]. Fan et al. [30] constructed and evaluated an individual’s Cardiorenal Syndrome Type 1 (CRS1) risk nomogram for patients with Acute heart failure (AHF). Demographic and clinical data were collected from the patient’s EHR, and a logistic regression model was applied for this study.
- **Mortality prediction**—For mortality prediction, four studies were reported in the literature [19,21,40,46]. Vignoli et al. [46] presented a study aimed to characterize the metabolomic fingerprint of acute MI using nuclear magnetic resonance spectroscopy on serum samples from patients and assess the potential significance of metabolomics in the predictive classification of acute MI patients. Multivariate statistics were used to build a predictive model for death within two years of a cardiovascular event. Finally, a prognostic risk model predicted death with a sensitivity of 76.9 per cent, a specificity of 79.5 per cent, and an accuracy of 78.2%, with an area under the receiver operating characteristic curve of 85% [46]. In [40], medical records were examined to evaluate the potential risk for patients undergoing transcatheter aortic valve implantation (TAVI). An extreme gradient boosting (XGBoost) model was utilized to investigate the impact of feature selection on the model’s performance. The authors compared machine learning models for all-cause mortality with traditional risk scores. Their results indicated that the machine learning model outperformed traditional risk scores and improved patient selection for all-cause mortality in the hospital. Medical records were consulted for the following information: patient’s demographics and medical conditions, results of tests and imaging studies such as electrocardiograms and echocardiograms, and reports from CT scans and MRIs [40]. Models for all-cause mortality were compared to risk scores that were used before new models were developed. An extreme gradient boosting (XGBoost) model was used to examine the effect of feature selection on performance. Lastly, the outcome of this study showed that machine learning was finally able to obtain significantly better results. Furthermore, it improved patient selection compared to older risk scores for "all-cause death" in the hospital [40]. A study evaluated the impact of age on percutaneous coronary intervention in a large, random sample of patients (PCI) [21]. Therefore, demographic data, clinical data, and procedural characteristics were collected from the patient’s EHR, and multivariate Cox regression analyses were applied. In [19], patients with coronary heart disease were evaluated using a variety of machine and deep learning models to predict five-year mortality rates. These models are the support vector machine, decision tree, random forest, gradient boosting, neural network, and logistic regression. Demographic and physical features, comorbid conditions, medication, laboratory biomarkers, and electrophysiological results were among the data modalities acquired from EHR in this study. Furthermore, only age, dyslipidaemia, prior cerebrovascular disease, and random forest score remained statistically significant in multivariate modelling, confirming their independence from the other factors [19].
- **Disease prediction**—Six studies were carried out [20,22,23,31,32,44] for disease prediction. Precision medicine was utilized in one study to discover risk polymorphisms in hypertension in African Americans that altered left ventricular mass linked with body surface area (LVMI) as a measure of cardiovascular disease risk by using a convolutional neural model [44]. Participants’ demographic information, past medical history, current medical condition, laboratory results, and CMR results are collected to evaluate LVMI [44]. The results showed that feature learning and representation produced better results than others [44]. One study [23] used machine learning approaches random forest model to develop a similar panel to predict incident coronary heart disease. Data from demographics, clinical and genetic data, and epigenetics were used in this study. This study reported a novel precision medicine tool based on DNA that is capable of capturing complicated genetic and environmental risk variables for CHD [23]. Another study gathered predictor factors from the EHR, knowing that they were routinely documented and accessible during the period examined [32]. The study used a regularized logistic regression model to predict 30-day readmission risks for heart failure, and the results can be used to determine patient risk for readmission and to guide clinicians in delivering precise health interventions. A study argued by Broers et al. [22] reported that patients with cardiac problems could improve their prognosis by altering lifestyle factors. Hence, the data modalities that were collected from EHR were demographic data and environmental lifestyle data, e.g., physical activity and sleep tracking. An analysis of the trajectories of outcome variables was performed using a locally weighted error sum of squares (LOESS). Predictors of both progress and deterioration in outcome measures were discovered using the linear mixed-effects regression technique [22]. A study was conducted to establish a foundation for more accurate, individualized risk assessment in individuals with chronic heart failure [31]. The data modalities were from EHR (demographics, clinical data, blood test, ECG) and echocardiography data and all these variables were entered into the multivariable Cox regression model [31]. Cine cardiac magnetic resonance (Cine-CMR) images are used for clinical diagnosis to differentiate between myocardial infarction (MI) and viable tissues/normal cases, where the support vector machine and logistic regression were applied in this study to predict coronary heart diseases [20].
- **Dose prediction**—Only one study was reported for dose prediction [24]. This study used demographics, clinical characteristics, and medical therapy as input data for regression models based on machine learning methods (random forest, boosted trees, linear regression, and optimal regression tree) [24]. The experimental results showed that data-driven models for customized coronary artery disease (CAD) management using electronic health records significantly improved health outcomes relative to the standard of care. In total, 81.5% AUC for each treatment has been achieved based on medical history and clinical examination results.

#### 3.4.2. Diagnosis

A diagnosis could be described as a process as well as a classification system or a set of pre-existing classifications used by doctors to identify a particular disease [47]. For diagnosis, 6 studies were identified out of a total of 28 most relevant articles. Alimadadi et al. [42] used omics data modalities by collecting RNA-Seq data to detect cardiomyopathy, which can present in two major clinical forms, dilated cardiomyopathy (DCM) or ischemic cardiomyopathy (ICM). This study showed the potential of using artificial intelligence via machine and deep learning models to diagnose cardiomyopathies with an improved level of precision. Five ML and DL algorithms were used in this study, which were the support vector machine with the radial kernel (svmRadial), neural networks with principal component analysis (pcaNNet), decision tree (DT), elastic net (ENet), and random forest (RF) [42]. The study [43] used echocardiography images and clinical variables to identify complex structural and functional abnormalities patterns in different cardiac pathologies using machine learning including the support vector machine and random forest model to integrate clinical and echocardiographic data. Moreover, a study used demographics, clinical data, and ECG singles from HER. In addition, coronary angiography image data were used to detect coronary heart diseases. Moreover, logistic regression was used in this study for the purpose of diagnosis [48]. MRI and electrocardiographic imaging were employed in another study to detect coronary heart diseases, and a fully convolutional network (FCN) was used to construct patient-specific 3D biventricular heart models from MR cine slices [26]. Similarly, Baessler et al. [27] used cardiac magnetic resonance (MR) images to determine whether texture analysis (TA) could be utilized to detect both acute and chronic myocardial infarctions. Texture features were used to distinguish between ischemic scar and normal myocardium using multiple logistic regression models. The results of this proof-of-concept study showed that TA of non-enhanced cine MR could accurately diagnose subacute and chronic MI [27]. For Aortic disease detection, one study was conducted using image clustering (CMR) to improve disease-specific treatment planning, risk assessment, and medical device development in complex diseases. A hierarchical clustering technique was used to gather subjects with similar characteristics, while issues with distinct differences formed another group [41]. 

#### 3.4.3. Phenotyping

Four studies contained information regarding phenotyping [28,29,37,38]. According to Zhao et al. [28], the dataset contained the electronic copy of each patient’s electronic health record. Before the first diagnosis, the dataset comprised 10 years of EHR data and consisted of phenotypic codes (PheCodes). This investigation used a non-negative constraint-tensor-factorization technique to extract phenotypic themes across time scales [28]. This data-driven approach is likely to support researchers’ efforts to identify complex and chronic disease sub-phenotypes in precision medicine. Study [37] reported the design for a prototype mechanism for atrial fibrillation (AF) warnings and evaluated the prototype’s efficacy and safety using an electronic health record (EHR) to gather demographic information, medical reports, such as clinical reports, radiology reports, CIED implantation reports, and lab test results. The AKENATON prototype workflow consisted of two steps: First, natural-language processing algorithms abstract the patient’s health record into the digital format, and second, an applied formal ontology-based and knowledge-based algorithm calculates and evaluates the patient’s anticoagulation status. Patients’ health records with similar clinical features were clustered or classified based on their similarity in patient similarity analysis. Based on echocardiographic features of left ventricular (LV) structure and function, hierarchical clustering techniques were applied to create a patient similarity network that predicted significant adverse cardiac events (MACE) in an individual patient [29]. Another study reported to provide individualized medication for patients with atrial fibrillation (AF) would be helpful in identifying sub-phenotypes (“endophenotypes”) of the condition. Demographic data and clinical outcomes were collected from EHR, and also echocardiography images were used in this study. A binary logistic regression analysis with univariate and multivariable options was performed to assess clinical associations for patients with AF [38]. 

#### 3.4.4. Risk Stratification

In the finalized 28 most relevant articles, four studies [33,34,36,39] reported their experimental results on risk stratification. Smole et al. [33] developed a new risk stratification model for human capital management using machine learning techniques known as HCM-RSS [33]. This works on patients’ current clinical status, imaging data, genetic data, and medical history to identify patients at risk of any severe adverse cardiac disease. This study involved different machine learning models (random forest, boosted trees, neural network, and support vector machine) that were fed with the patient data that were already mentioned. For each prediction, the risk stratification model explained the patient’s classification. Ref. [39] evaluated the risk of life-threatening ventricular tachyarrhythmias. Data were collected from known cases of chronic heart failure, including physical assessment, lab investigation, echocardiography (Echo), Holter monitoring (HM), stress test, and demographic data. The Cox regression analysis model was used. The study’s findings led to developing a new two-step paradigm for customized risk stratification in individuals with CHF. The classification model had an 80.8 per cent sensitivity and 99.1 per cent specificity. Finally, Individualized risk assessment algorithms based on logistic regression models correctly classified 93.9 per cent of CHF patients. Moreover, an experiment was performed to demonstrate how an outcome-driven strategy could be used to detect clinically similar individuals [36]. As a result, patients with comparable clinical outcomes were likely to be classified together [36]. An atrial fibrillation patient cohort currently thought to be at high risk of an ischemic stroke (IS) was used in a real-world case study. A hierarchical clustering, agglomerative clustering, was used to group patients in a comparable context. It began with a single cluster and merged the two “closest” clusters at each stage until the process was complete. Finally, the method was able to identify a precise group of patients with a low probability of developing IDS.

Lastly, a study was conducted to determine the probability of sudden cardiac death (SCD) or pump failure death (PFD) in chronic heart failure (CHF) patients; models based on clinical characteristics were developed [34]. This study aimed to determine whether merging standard clinical factors with ECG markers for autonomic nervous system (ANS) imbalance and electrophysiological abnormalities would increase the capacity to stratify SCD and PFD risks. The Cox regression was used to determine whether each potential risk marker was associated with SCD or PFD [34]. Finally, a risk model constructed entirely from conventional clinical characteristics could significantly enhance the prediction of syncope and pump failure events in patients with chronic heart failure.

### 3.5. Artificial Intelligence Algorithms

After analysing the literature, it was concluded that most of the research models used traditional classification models. No advanced or hybrid artificial-based models were reported in the extant literature. The ML-based models reported in this research domain are depicted in Table 2.

**Table 2:** Detection of different types of CVD using AI-based techniques.

S. No	AI-Based Models	References
1.	Logistic Regression	[19,20,24,27,30,32,33,38,46,48]
2.	Random forest	[19,23,24,33,40,42,43,46]
3.	Support Vector Machine	[19,20,33,35,40,42,43]
4.	Neural Network	[19,26,33,42,44]
5.	Clustering (Hierarchical clustering)	[36,41]
6.	Cox regression	[21,22,31,34]
7.	Gradient boosting	[19,25,33]
8.	Decision Tree	[19,42]
9.	Locally Weighted Error Sum of Squares (LOESS)	[22]
10.	Tensor-Factorization	[28]

### 3.6. Datasets

Different data modalities (datasets) were reported in the studies, such as EHR, image, omics, lifestyle, and environmental data. In the articles [19,21,22,23,24,25,28,29,30,31,32,33,34,35,36,37,38,39,40,44,48], EHR data were used for the experimental and identification process. It comprised demographic data, physical characteristics, medication, laboratory results, medical history, patient vital signs, and procedure characteristics if the patient had a procedure related to the heart. For cardio-relevant problems, three types of MRI images [20,26,27,41,44], CT scan [40], and echocardiography [26,29,31,33,39,43,48] images were used for the early prediction and diagnosing process. In lifestyle and environmental data, Broers et al. [22] used sleep tracking, physical activity, and consuming alcohol. The omics data [23,25,33,42] were another big database tool used for disease diagnosing and prediction purposes. It mostly comprised the proteomics and RNA-Sequence data.

## 4. Discussion

This section of the paper outlines the key findings of this research work. Moreover, it briefly outlines the implications of this scoping review work.

### 4.1. Principal Findings

In this review process, the explication of artificial intelligence was reviewed for precision medicine in cardiovascular medicine. From a total of 697 retrieved studies, 28 were included in this review. The included papers were reported in the years ranging from 2015 to 2022. Among the countries that contributed to the domains of precision medicine, the United States has reported more research trends for precision medicine in cardiovascular medicine, with a total of 16 papers.

The findings of this scoping review were classified into three broad categories, where each category represented a different classification of the reviewed papers. The first category focused on the cardiovascular branches. It was further divided into six subcategories: coronary heart disease, arrhythmia, heart failure, aortic disease, cardiomyopathy, and hypertension. The second category was the precision medicine branch, and it contained four subcategories: predicting, diagnosing, risk stratification, and phenotyping.

The third category was based on the AI algorithms used in the targeted research trends. These AI-based algorithms were classified into eleven subbranches. The most common algorithms used were logistic regressions, random forests, support vector machines, and neural networks. For the AI algorithms used in the studies, we discussed the type of algorithm used in the studies. Moreover, the algorithms proved their ability to predict and classify diseases based on the data used to train the different models.

### 4.2. Practical and Research Implications

This review process highlighted commonly used AI models for precision medicine in cardiovascular diseases. Based on our findings, AI models have shown promising performance in various branches of precision cardiovascular medicine, as reported in most studies. One possible explanation is that AI and machine learning algorithms can process large amounts of patient data from multiple sources, including electronic health records, mobile healthcare applications, and medical images [49]. This enables the development of more accurate and personalised predictive models for cardiovascular disease, which can identify early warning signs and risk factors before symptoms appear. Another potential factor is the ability of AI and machine learning algorithms to detect subtle patterns and relationships within the data that may not be visible to the human eye. For example, CNNs have been shown to be effective at analysing medical images, such as CT scans and MRI scans, to detect abnormalities and predict outcomes [50]. Similarly, gradient-boosting classifiers and random forests can identify important features and relationships within complex datasets, which can then be used to make accurate predictions. Furthermore, AI and machine learning algorithms can be trained and updated continuously as new data become available. This allows for real-time monitoring and prediction of changes in a patient’s condition, which can lead to more timely interventions and improved outcomes. Overall, the use of AI in precision cardiovascular medicine has shown great promise and offers many potential benefits for patient care.

To be clear, none of the listed ML applications was created to take the role of a therapist but rather to improve the physicians’ abilities and the quality of treatment they provide. Since there are not many scoping reviews performed in this field, a more focused scoping review is required to address the use of AI for precision medicine based on the purpose that was discussed in this paper.

#### Strengths and Limitations

We were able to capture evidence about the successful ML algorithms that have proven their capabilities to predict risk for cardiovascular diseases or predict risk and disease complication and mortality rate. To the best of our knowledge, there was no scoping review discussing precision medicine in cardiovascular diseases in general; the other review discussed the AI used for specific purposes, such as predicting cardiac arrest. For that reason, this scoping review is the first one to conduct and explores the different algorithms used in this field of precision medicine in CVD.

This review has some limitations. We limited our review to articles published in the English language. As a result, we may miss some studies.

## 5. Conclusions

This scoping review aimed to investigate the use of AI models in precision medicine for CVDs. We examined various branches of precision medicine and machine learning algorithms using patient data from various sources to predict the risk of heart diseases such as coronary artery disease, arrhythmia, and heart failure. The findings from the most relevant articles suggest that precision medicine can improve the diagnosis and prediction of various cardiovascular diseases. Furthermore, the availability of a large number of patient data from sources such as electronic health records and mobile healthcare applications allows for the development of new algorithms that can reduce mortality rates, improve the quality of life for cardiac patients, and predict the risk of diseases. These advancements have the potential to impact the health of individuals and communities.

## Figures and Tables

**Table 1:** The demographics of the studies included in the analysis.

Characteristics	Number of Studies Included
Publication type	
Journals	*n* = 26
Conference	*n* = 1
Books	*n* = 1
Country	
United States of America	*n* = 16
United Kingdom	*n* = 5
China	*n* = 3
Netherlands	*n* = 2
Amsterdam, Australia, Belarus, France, Germany, Iran, India, Italy, Slovenia, Switzerland	*n* = 1
Year of publication	
2020	*n* = 5
2021	*n* = 5
2022	*n* = 1
2018	*n* = 5
2019	*n* = 2
2017	*n* = 6
2016	*n* = 2
Sample size	
<1000	*n*= 16
1000–20,000	*n*= 7
>20,000	*n*= 4

## Supplementary Materials

The following supporting information can be downloaded at: https://www.mdpi.com/article/10.3390/jpm13081268/s1, Supplementary File S1 and Supplementary File S2.
