# Supplementary material for: Artificial Intelligence-Based Methods for Precision Cardiovascular Medicine
Source: J Pers Med. 2023 Aug 16;13(8):1268. doi: 10.3390/jpm13081268 (PMC10455092; doi:10.3390/jpm13081268)
Supplement: Supplementary file 1 [file jpm-13-01268-s001.zip › jpm-2187146-Supplementary File S1.pdf]

Search history

Search string	Database	# Of studies	Date
(TITLE-ABS-KEY ("machine learning") OR TITLE-ABS-KEY ("deep learning") OR TITLE-ABS-KEY ("artificial intelligence") OR TITLE-ABS-KEY ("neural network*") OR TITLE-ABS-KEY ("Convolutional neural network*") OR TITLE-ABS-KEY ("reinforcement learning") OR TITLE-ABS-KEY ("transfer learning") OR TITLE-ABS-KEY ("supervised learning") OR TITLE-ABS-KEY ("unsupervised learning") OR TITLE-ABS-KEY ("ensemble learning") OR TITLE-ABS-KEY ("Support vector machine") OR TITLE-ABS-KEY ("Artificial neural network*") OR TITLE-ABS-KEY ("decision tree") OR TITLE-ABS-KEY ("Random Forest") OR TITLE-ABS-KEY ("Naïve Bayes") OR TITLE-ABS-KEY ("Naive Bayes") OR TITLE-ABS-KEY ("K- Nearest Neighbor*") OR TITLE-ABS-KEY ("K-means") OR TITLE-ABS-KEY ("Long Short-Term Memory Networks") OR TITLE-ABS-KEY ("regression analysis")) AND (TITLE-ABS-KEY ("precision medicine") OR TITLE-ABS-KEY ("personalized medicine") OR TITLE-ABS-KEY ("individualized medicine")) AND (TITLE-ABS-KEY ("cardiovascular disease*") OR TITLE-ABS-KEY ("stroke") OR TITLE-ABS-KEY ("heart disease*") OR TITLE-ABS-KEY ("aortic stenosis") OR TITLE-ABS-KEY ("aortic disease*") OR TITLE-ABS-KEY ("coronary arteries disease*") OR TITLE-ABS-KEY ("hypertension") OR TITLE-ABS-KEY ("atrial flutter") OR TITLE-ABS-KEY ("heart failure ") OR TITLE-ABS-KEY ("atrial fibrillation") OR TITLE-ABS-KEY ("cardiomyopathy") OR TITLE-ABS-KEY ("rheumatic Heart Disease") OR TITLE-ABS-KEY ("cardiology")) AND (LIMIT-	Scopus	474	30 Jan 2022

TO (PUBYEAR , 2022) OR LIMIT-TO (PUBYEAR , 2021) OR LIMIT-TO (PUBYEAR , 2020) OR LIMIT-TO (PUBYEAR , 2019) OR LIMIT-TO (PUBYEAR , 2018) OR LIMIT-TO (PUBYEAR , 2017) OR LIMIT-TO (PUBYEAR , 2016) OR LIMIT-TO (PUBYEAR , 2015)) AND (LIMIT-TO (LANGUAGE , "English"))			
(("precision medicine"[Title/Abstract] OR "personalized medicine"[Title/Abstract] OR "individualized medicine"[Title/Abstract]) AND ("regression analysis"[Title/Abstract] OR "machine learning"[Title/Abstract] OR "deep learning"[Title/Abstract] OR "artificial intelligence"[Title/Abstract] OR "convolutional neural network*"[Title/Abstract] OR "reinforcement learning"[Title/Abstract] OR "transfer learning"[Title/Abstract] OR "neural network*"[Title/Abstract] OR "supervised learning"[Title/Abstract] OR "unsupervised learning"[Title/Abstract] OR "ensemble learning"[Title/Abstract] OR "Autoencoder"[Title/Abstract] OR "support vector machine"[Title/Abstract] OR "naive bayes"[Title/Abstract] OR "decision tree"[Title/Abstract] OR "random forest"[Title/Abstract] OR "naive bayes"[Title/Abstract] OR "k nearest neighbor*"[Title/Abstract] OR "K-means"[Title/Abstract] OR "long short term memory networks"[Title/Abstract]) AND ("Cardiovascular"[Title/Abstract] OR "stroke"[Title/Abstract] OR "heart diseases"[Title/Abstract] OR "aortic stenosis"[Title/Abstract] OR "aortic diseases"[Title/Abstract] OR "coronary arteries diseases"[Title/Abstract] OR "hypertension"[Title/Abstract] OR "atrial flutter"[Title/Abstract] OR "heart failure"[Title/Abstract] OR "atrial fibrillation"[Title/Abstract] OR "cardiomyopathy"[Title/Abstract] OR "rheumatic heart disease"[Title/Abstract] OR	Pubmed	105	30 Jan 2022

"cardiology"[Title/Abstract])) AND ((medline[Filter]) AND (2015:2022[pdat]))			
"cardio*" And "precision medicine" And "AI"	Google scholar	100	30 Jan 2022
